# Southern Dental Association

**Published:** 1888-02

**Authors:** 


### PROCEEDINGS.

### Southern Dental Association.—Nineteenth Annual Session, 1887.

REPORTED FOR DENTAL REGISTER BY MRS. M. J. W.

The Southern Dental Association held its Nineteenth Annual Session at Old Point Comfort, Va., in the Hygiea Hotel, where most ample accommodations were provided for sessions, clinics, displays, and the social features of the meeting.

The Association was called to order at 10 A. M., Tuesday, August 30, by the President, Dr. W. W. H. Thackston, of Farmville, Va., who in a few well-chosen words, bade all present welcome to Virginia. Dr. J. H. Prewitt, of Madisonville, Ky., responded in a most eloquent address, justifying his right to the title, which has been bestowed upon him, of the Demosthenes of the dental profession.

Dr. V. E. Turner, of Raleigh, N. C., welcomed those present as guests of the Southern Dental Association.

Dr. J. Taft, President of the Section of Dental and Oral Surgery of the International Medical Congress, was introduced by the President, and made a few remarks inviting all dentists present to participate in the proceedings of the Congress.

Dr. Geo. H. Forney, U. S. Army, Surgeon of Fortress Monroe, was introduced. To him the Association was indebted for a corps of soldiers as subjects for the clinics.

Many visiting dentists were present, among them Drs. Mc Leod, of Edinburg; Wedgewood and Crowley, of London; Thomas, of Paris; Sjoberg, of Stockholm; also Drs. Younger, of California; Baldwin, Harlan, Allport, Taft and others from the West; Atkinson, Pierce, Shepherd, Walker, Bonwill, Stockton and others from the East. The privileges of the floor were tendered to all visiting dentists.

Prof. J. B. Hodgkins, of Baltimore, offered the following resolutions:

*Whereas*, this Association has learned with deep regret of the death of Dr. J. R. Walker, of New Orleans, one of its oldest and most active members: *Resolved*, that a Committee of three be appointed to draft suitable resolutions expressive of the sense of loss this Association has sustained, and present them at a suitable time for consideration.

*Resolved*, That a memorial page be set apart on the roll-book of this Association, to be inscribed with the name of the deceased member.

The resolutions were unanimously adopted, and a committee of three appointed.

Dr. A. E. Baldwin (Chicago) having prepared a paper for the association, by special request, was appointed to open the morning session Wednesday, August 31. His paper was entitled:

IMMEDIATE ROOT FILLING

of which a brief abstract follows:

Although he did not condemn pulp-capping, he believed that as a rule the better plan was to devitalize, the usefulness of the pulp having been mainly accomplished when the tooth is fully developed; he would therefore make greater efforts to save the pulp alive in the tooth of a child than in the adult.

Teeth with abscessed roots may, as a rule, be immediately filled, after thoroughly cleaning out and drying. The canal may be wiped out with a 95 per cent. solution of carbolic acid, followed by alcohol, but the main part of the treatment consists in thoroughly drying the canals and tubules, to the point of dessication, with hot air. When this is thoroughly done, the material used for filling is indifferent, provided that it be such as will seal the apex and tubuli, and be non-irritating, The writer of the paper uses base-plate gutta-percha in chloroform, of the consistency of cream; when the root canal is thoroughly filled, by pumping in by means of shreds of cotton wrapped on a broach—the latter made of fine piano-wire roughened by rolling on hard wood under a fine file—a small piece of heated gutta-percha is placed over the opening, and with fine-pointed instruments, the contents are forced into all the interstices.

If the pulp requires devitalization he waits from a week to ten days before removing the dead tissue, that it may slough from the living tissue. The writer of the paper thinks that the importance of micro-organisms has been greatly exaggerated, and that pus is formed without their presence, as in the case of a felon, or synovitis of the knee joint. He does not think a long list of remedies necessary but that a few, carefully selected will meet all requirements.

Dr. M. C. Marshall (Little Rock, Ark.) read a paper on

CONSERVATISM IN THE SELECTON OF FILLING MATERIALS.

He said that he who would best serve his patients must adopt a conservative course, and should have the courage of his convictions. It is mechanical to introduce the material properly; artistic to reproduce nature; but scientific to determine what is best in any given case. He must consider the constitutional tonicity, and the age of the patient. Duty must transcend any hobby, and the æsthetic must not dominate the practical.

These papers were discussed at considerable length.

Dr. Winckler (Agusta, Ga.) thought the paper contained many valuable suggestions, especially in regard to the thorough drying of the canals and dentine. He preferred lead points or carbolized wood for root filling. He devitalizes by knocking out with the wooden peg, a method which is almost painless when skillfully done. Dormant dead pulps when opened into, he treats with peroxide of hydrogen until ebullition ceases; he then wipes out thoroughly and fills with iodoform and carbolic acid paste, waiting at least a day before permanently filling.

Dr. Storey (Dallas, Texas.) said that from what had been said one might suppose all root canals were as big as gun barrels and as straight, but they were not made that way in Texas; there were many that were so crooked and so small that it was impossible to get any thing either in or out. He uses oxy-chloride of zinc every time, and does not know that he has lost a case. He wanted the brethren to tell him more about those drills that have sense enough to go around corners, and stop when they get to the end of the root.

Dr. L. G. Noel (Nashville, Tenn.) was a profound skeptic as to the necessity of such absolute dryness of the canal; he did not believe they would stay dry; the fluids would find their way back through the tubuli by capillary attraction.

Dr. C. E. Kells, jr., (New Orleans) said that if the root filling was perfect there would be no danger from moisture, as both the fluid and the filling could not be in the same place. If a root canal was so fine that a nerve bristle could not enter, it did not need filling. The best filling for canals, in his opinion, was carbolized osage wood. He had long since abandoned the useless method of long treatment. The cause of trouble is the dead pulp, and that can be as thoroughly removed in one sitting as in repeated attempts. Abscess can be as well treated after as before filling the root.

Dr. J. R. Knapp (New Orleans) did not think wood best unless the canals are large and round. Fluid and solid materials should be used together, the solid filling crowding the fluid into all the interstices.

Dr. W. H. Morgan (Nashville, Tenn.) said that the trouble from devitalized teeth is the result of putrefaction, and any thing that will prevent putrefaction will prevent abscess; if dessication can be accomplished decomposition will be obviated; though he should continue to rely on antiseptics he would use hot air as an adjunct. He took issue with Dr. Baldwin's method of root filling as liable to force the material through the apex where it would create inflammation. He did not think the tubuli could be so sealed but that fluids would enter by endosmosis.

Dr. T. T. Moore (Columbia, S. C.) did not believe there were any drills that could follow the contortions of some roots; there were many that could not be filled at all. As filling he used liquid gutta-percha into which he drives a point of either gutta-percha, lead, or wood. He wished to ask Dr. Winckler if the operation of driving a peg into a living pulp was not very painful?

Dr. Winckler replied that until tried it was impossible to believe how entirely satisfactory the method is to both patient and operator, and how little sign of pain is evinced by the patient.

Dr. W. H. Richards (Knoxville, Tenn.) when forced to devitalize, applies arsenical paste, and waits only four hours, when the pulp will be found in a congested but anesthetized condition when it can be removed with but little pain. After removing the pulp he fills with a paste of carbolic acid and iodoform to prevent septic formations in the tubuli.

Dr. Sjoberg (Stockholm, Sweden.) described Witzel's method, largely used in Sweden and Germany.

The pulp is cauterized with arsenical paste and the coronal portion removed with a clean new bur, a small pellet of oxychloride paste being placed over the openings to the root canals and covered with a platinum cap, over which the pulp chamber is filled.

Dr. H. J. McKellops (St. Louis, Mo.) had yet to see the man who could honestly say he made an absolute success of root filling. Often after a man has done his very best an abscess will form, and we cannot say when it will happen, or why.

Dr. H. E. Beach (Clarksville, Tenn.) would enlarge the canal sufficiently to make it round and ready for a metallic filling as he used lead for root fillings being thoroughly satisfied with it after an experience of seventeen years.

Dr. Geo. Evans (New York City) asked attention to an instrument he had devised for drying out canals, and to allay sensitiveness of dentine, which he would exhibit at the clinics.

Dr. McKellops commended the gold broches introduced by Dr. Herbst.

Dr. A. Eubank (Birmingham, Ala.) dries out with chloroform.

Dr. C. S. Stockton (Newark, N. J.) does not believe in immediate root filling; prefers a temporary filling, the patient losing nothing by waiting.

Dr. W. W. Allport (Chicago) while desiring to save the pulp alive when possible, fills immediately when the pulp has to be removed. To remove the contents of the tubuli and put them in a healthy condition, he uses a heated wire with which to dry out the canals, after which he injects peroxide of hydrogen until no more gas is evolved. He uses oxychloride of zinc in semi-fluid form, filling the chamber full. He objects to gutta-percha as liable to shrinkage and porocity.

Dr. R. R. Freeman (Nashville, Tenn.) advised all who had not used lead for root fillings to go home and try it during the coming year, and report at the next meeting. He tried to preserve all the pulps possible; when that can not be done, he lets them die as easily as possible.

At the afternoon session Dr. Staples (Sherman, Texas) read a paper on

### CAUSES OF FAILURES IN FILLINGS.

He attributed ninety-five per cent. of all failures to *lack of thoroughness* at some point, beginning with the selection of material out of which to make the dentine. Some men are born slipshod, some are born stingy, some are born cranky, and neither class will be thorough in any business. We want thoroughness at every point, and at every step; a thorough application of the instrument at one end, and a thorough dentist at the other; this alone will insure success.

Dr. T. H. Parramore (Hampton, Va.) read a voluntary essay on

### ASEPTIC SPONGE FOR PULP GRAFTING.

He said that in the treatment for disease we too often disregard hints given us by our great teacher, Nature. Man is but an aggregation of cells, with tissues built up by the white blood corpuscles, under proper environments transformed into bone, muscle, teeth, hair, etc. Millions of microscopic bodies dance in every drop of water, never becoming anything more, except through change of environment. As this spawn only requires protection and assistance to reach its highest development, might not the same be predicted of the white corpuscle? That such is the fact has been demonstrated in what is known as "Sponge Grafting." The increased flow of blood to a point of irritation being for the purpose of repair or tissue building, the surgeon or dentist who renders nature most efficient aid in this effort is most nearly fulfilling the purpose of his calling. "Pulp stones" are evidently an abortive attempt at self-protection. Can we aid Nature in placing these deposits at the point of exposure? The irritation of an exposed pulp causes an increased flow of blood bringing the white blood corpuscles for the work of repair; a bit of aseptic sponge placed exactly at the proper point, seizes upon these corpuscles, cements them with fibrin, nourishes them with protoplasm until the whole becomes an adamantine barrier behind which the pulp rests secure. Having used aseptic sponge exclusively in capping exposed pulps, the author of the paper suggests it to the profession as a method worth investigating. The cavity is prepared as usual, being careful to wound the pulp as little as possible; it is swabbed out with cotton, saturated with a $\frac{1}{500}$ solution of bichloride of mercury, and dried, the aseptic graft being placed dry directly upon the pulp, and the cavity filled with oxyphosphate.

Dr. G. H. Winckler (Augusta, Ga.) read a paper on

### Soft Gold Foil.

Meaning by this term gold, which by some process, in manufacturing into foil has been deprived of that inherent property of chemically pure gold, freshly annealed, of welding or cohering when pressed or malleted together. In the paper read the author said that he wished to elucidate his appreciation of the value of soft gold foil in the preservation of teeth, the character of the operations in which its use is indicated, and his own method of using it, (which he also demonstrated in his clinics). He spoke of fillings inserted thirty and forty years ago, before cohesive gold was known, and which are still preserving the teeth, the cavities being entirely free from decay, though some of the filling are so soft that an excavator might be pressed through them. In his own practice he uses soft gold, cohesive foil, and the two combined, as his judgment dictates, finding nothing so entirely satisfactory as the former, in the cavities where its use is indicated. It is never used except honestly, for the preservation of teeth, as it makes no show; it can be inserted with the greatest ease, and rapidly; is readily adapted to the walls of the cavity; it makes a filling sufficiently compact for the service required of it, and as it condenses laterally under pressure it protects the cervical walls. It enables the operator to economize both time and labor, and thus protects his patients from fatigue. It always does away with the necessity for using the rubber dam so objectionable to many. Its habitual use develops manual dexterity and mechanical ingenuity, as success depends upon the mechanical principles of mortising and dovetailing. The author of the paper considers soft foil unquestionably best in all simple cavities where walls are intact, in cavities at or under the free margins of the gums, in simple proximal cavities not requireing to be knuckled up. Proximal cavities that require to be contoured, he would fill to at least two-thirds their extent with soft foil, welding on cohesive gold, making undercuts if necessary, for the contour portion. The remarkable strength of the union of cohesive gold welded to soft is incredible to those who have never used the combination. He uses pellets almost exclusively, taking a small piece of foil torn from the leaf and folded on itself tightly, first in one direction and then across. making a loose mat which he crumples and rolls between his fingers and thumb making one end rather pointed. The crumpling makes little wrinkles which, under pressure, interdigitate and make a solid mass of great tenacity. He depends on under cuts, but no remaining pits. In inserting the pellets he inserts the last against the side of the cavity where it slips in more readily than between two surfaces of partly condensed gold. He uses smooth points and carefully avoids puncturing the foil; he uses hand pressure and the lead mallet, but depends npon plugging forceps for condensing the gold, those for condensing in crown cavities in lower molars bearing on pad placed outside of the mouth under the jaw, the outer teeth forming a condensing point. He uses

No. 3, 5 and 6 gold foil and carefully selects his foil. He thinks that every operator should perfect himself in the art of using both forms of gold; he who cannot do so depriving both himself and his patients of many advantages.

Dr. J. B. Hodgkin, (of the Baltimore College of Dental Surgery) next read a paper on

AMAGAMS AND METHODS OF USING.

He said that entering upon the Amalgam Controversy was like a song too often repeated, yet there might probably be a fresh presentation of its old aspect.

The science of making and working amalgams is based upon the occult, hidden laws of metallurgy—a science built upon what we assume to be from the little we see.

Though we might suppose like causes would produce like effects, yet we cannot predict the behavior of an alloy from a knowledge of its constituents.

Prof. Hodgkins then cited some of the curious results of the mixing of different metals and the still more curious results from the introduction of mercury, the most brilliant imagination being incompetent to picture the molecular arrangement, deducing the conclusion that with all we know of amalgam, it is still more or less a mysterious subject. As the practical results of years of observation he is convinced that amalgam in proximal cavities lacks the elements of permanent tooth saving, and offers "a Norfolk oyster supper, with fixin's" to any one who will bring him a case of two proximal cavities in molars or bicuspids, encroaching on the gum, which were filled with amalgam five years ago, which are not now leaking, and are still preserving the teeth.

Though many a tooth is saved for a time by amalgam that would otherwise "have gone to the shades," yet, of all fillings, time is needed as a test of merit of amalgam.

The points raised in these papers, and the general subject of Operative Dentistry were discussed until the hour for adjournment. Nothing new was elicited, the discussion being limited mainly to the expression of individual preferences.

THURSDAY, Sep. 1, was devoted to clinics.

Dr. Younger (San Francisco) gave two clinics in Implantation; an inferior central incisor for a soldier from Fortress Monroe, the natural tooth having loosened and been removed with the fingers some months previous, extensive absorption having taking place. The adjacent teeth had approached each other so much that it was necessary to cut them away with corundum disks to make place for the tooth to be implanted.

The other case was a superior bicuspid implanted for Dr. D. D. Lester, of Christiansburg, Va. In reply to questions he stated that the operation of forming the artificial socket was less painful than the usual preparation of a cavity for filling.

Dr. A. E. Baldwin (Chicago) demonstrated his method of immediate-root-filling, and treatment with hot air, the tooth being a superior second bicuspid, of which Dr. B. S. Byrnes (Memphis, Tenn.) subsequently filled an approximate crown cavity. Dr. Brynes also filled, with soft foil in ribbons, a buccal cavity in an inferior, left, third molar, working under difficulties, having only a broken excavator to work with and using bibulous paper instead of rubber dam. Dr. Brynes used in these clinics the working model of his new automatic mallet the special feature of which is its ready attachment to any hand-piece by a simple attachment like putting a bit in a stock with a half turned clamp. The force of the blow is controlled, or entirely checked, at will, by a spring under the index finger and depends on the weight of the hammer rather than the force of a spring.

Dr. W. N. Morrison (St. Louis) filled tortuous roots with his well-known skill.

Dr. J. G. Morey was present with his nerve-drills, drilling roots and proving their great value in the preparation of root canals for the reception of pins for crown or bridge work.

Dr. Morgan (Staunton, Va.) in filling a labial cavity demonstrated the merits of Dr. D. B. Freeman's new double-loop spring clamps, which hold the rubber dam in position and crowd the gum back at the cervical border.

Dr. Geo. Evans (New York) inserted one of his seamless-gold-contour crowns made at the chair. The crown was made and the roots prepared ready for cementing in twenty-five minutes.

Dr. Evans also exhibited his "bulb and broach" instrument for drying out the canals and tubuli. The instrument consists of an oval ream of silver which, being heated in the flame of the alcohol lamp, retains the heat and transmits it to the tooth through the broach.

Dr. Evans also exhibited specimens of his removable bridge-work, porcelain fronts for gold crowns, etc.

Dr. Genese (Baltimore) gave several clinics in prosthetic dentistry, using his own articulator upon which he modeled several cases consecutively, and using his new pinless teeth in two complete upper dentures. He also demonstrated his method of packing without having any excess of rubber, and Richel's Automatic Vulcanizer. He exhibited and demonstrated the value of his "syphon tongue-holder" and his "speculum and cheek-distender."

Dr. L. P. Dottiner (Charleston, S. C.) restored a superior bicuspid with an all-gold crown, made at the chair.

Dr. H. A. Parr (New York City) crowned two central incisors which had been broken off seven years ago, and over the abscessed roots of which a plate had been worn, the gums being very unhealthy. One of the roots was split to the apex and had to be banded. The roots were treated and filled and the porcelain-front gold crown placed in position, the whole operation being completed in about two hours.

Dr. Parr also applied his "Universal Separators," obtaining sufficient space for thorough examination or operations, in less than three minutes in every case.

The displays made by the dental manufacturing companies were very complete, not only in all the regular lines of goods but in the number of new inventions and recent patents.

The S. S. White Co., the Welch Dental Co., the American Dental Manufacturing Co., Gideon Sibley, and others had fine exhibits.

Adjourned.

THURSDAY, 8. P. M., the Association was called to order, and Dr. E. R. Beadles, Danville, Va., read a paper entitled

### Dentistry. The Old and the New.

This was a general review of the history of the profession, and the advances made in the last quarter of a century; its rise from the *trade* to which men turned as a last resort when they were fit for nothing else, to a field worthy the talent of men of brains and intellectual capacity such as would fit them for success in any department of life. What was then considered degraded and lowly is now honorable and noble; empiricism and imposture have been replaced by skill and science. Dentists have gained the confidence of the intelligent by proving themselves intelligent until dentistry is now recognized as a specialty of medicine, its proper and legitimate place.

Among the obstacles still to be overcome are the "old fogies" to whom bridgework is a humbug; contour fillings worthless; the dental engine a curse, and the rubber dam only fit to be reversed in name; to whom wedging is barbarous; the use of the hypodermic syringe to be condemned as stabbing a man, etc. Hope lies in the fact that they cannot live forever.

Dr. Winckler replied to some points made in the discussion of his paper, and the subject of Operative Dentistry was then passed.

A paper from Dr. Hilgin (Jackson, Mississippi) was, in the absence of the author, read by title

### Mechanical Dentistry.

Dr. W. D. Dunlap (Selma, Ala.) read a paper on

### Dental Hygiene. A Study That Belongs to the People.

The writer believed this subject to be one of the most important, because upon its teachings depend the preservation of the teeth in a normal condition. It is one in which the people, both old and young, are vitally interested, and yet of which the masses are very ignorant. The writer believed that the day is at hand when text books should be prepared to be used in schools, giving plain hygienic rules covering the subject. The principles of dental hygiene should be carefully taught, and teachers, who now require clean face and hands, should also see that the mouth and teeth are also properly cleansed daily. Proper habits in this regard, formed in youth, will be retained in after years. More good could be accomplished in that way than by the whole profession with drills, burs, chisels, scrapers and pluggers. When the people are well versed in hygienic principles dental services will be called for in more timely season and better results will ensue than when work is delayed until trouble sets in. The services of Legislatures and School-Boards should be invoked to make such teachings compulsory.

This paper was discussed at considerable length.

Dr. R. Finley Hunt said that cleanliness could not be limited to one point, as *cleanliness*, as indicated in the paper read, or to the preparation of the Cereals, as expounded by Dr. John Allen, or to furnishing abundance of lime-salts to mothers and children as held by our esteemed and lamented brother from New Orleans, Dr. J. R. Walker, food-stuff in itself is not of so much importance as its assimilation. The Native Indians, the Esquimaux, the Sandwich Islanders—all had remarkable teeth, and yet their food habits varied widely. He hoped this would be made the subject of earnest study and investigation.

Dr. W. N. Morrison (St. Louis) said that much devolved upon the dentist in this line. The best work that we can do falls into ruin for want of proper care. He thought that the training of children to proper habits in this respect was of the greatest importance; if the deciduous teeth are properly cared for, the second teeth will be benefitted by it. He thought we had gone to extremes in the prepartion of our food, doing away with mastication.

Dr. Storey spoke of the proverbial *tough beef* of Texas and the correspondingly strong fine teeth of the Texans, also of the abundant deposits of tartar on the teeth in the " black lands," or gypsum regions.

Dr. W. H. Morgan thought that in the study of Hygiene, the object should be to attain the highest standard of health, and the power to resist the encroachments of disease.

Dr. J. T. Crawford (Nashville) considered this subject as one of the first importance on the grand prophylaxy. If the line indicated in the paper could be followed out we would accomplish much for the profession and for the people. Proper care of the teeth, backed by dietetic care might bring back the condition of the teeth of the aboriginies, though this did not imply a return to their barbarism. This subject is of vast importance, and far-reaching in its influence over those yet unborn, and through which we may confer lasting benefits upon the human family.

Dr. R. Finley Hunt said, that in the case of his own children where every possible care had been given, it had availed but little, probably through lack of assimilation, to have perfect teeth, it was necessary to have the whole system in perfect condition. proper food, taken at proper times, with exercise in the fresh air and sunlight did not imply barbarism. To bring the human system to perfection of assimilation is the highest point of dental hygiene.

Dr. J. B. Hodgkins (Washington) said that constitutions were laid down on certain lines, with a trend or type of hair, eyes, etc., and also of teeth ; those lines could not be stamped out.

Dr. W. H. Atkinson (New York) said that the forces that move men and map out their career were at work before this planet had any inhabitants. Hygiene means health ; in health each part does its whole duty ; a knowledge of embryology was necessary to a full comprehension of the subject.

Dr. Friedrichs (New Orleans) thought that though the original stamp might be the same, we had power of control of development—one might be starved and neglected—another well-fed and sheltered, bringing about very different finalities.

Dr. Harlan (Chicago) appeared on the part of the American Association, with reference to a joint meeting of the American and the Southern Associations.

A committee was appointed to confer upon the matter. They reported on Friday morning in favor of a joint meeting to be held in Louisville, Ky., in August, 1888. The report was accepted and adopted. A telegram was then read from Dr. Frank Abbott, President elect of the American Association, congratulating the Southern Association on its successful meeting.

Prof. Hubbard, Dean of the Dental Department of Meharry College, Tenn.. spoke on behalf of this Institution for the higher education of colored youths.

Prof. J. B. Hodgkins then read the following report from the committee appointed to draft resolutions in memory of the late Dr. J. R. Walker, New Orleans, La.

Dr. J. R. Walker was born in N. Y. State in 1830; was educated in Ohio; studied dentistry in the private offices of several dentists; settled in New Orleans in 1854, where he practised continuously (except during his service in the Confederate army). He was twice Vice-President of the American Dental Association, President of the Southern Dental Association, and was President elect of the National Dental Association at the time of his death.

He had been a Fellow of the American Association for the advancement of science, and Vice-President of the New Orleans Academy of Science, and a frequent contributor to scientific and dental journals.

He died at his summer home in Bay St. Louis, Miss., June 22nd, 1887, leaving a widow and five children.

Such was the man, who, were he still alive, would stand among us to-day. But, to read his career, as we should, we must needs go to the unwritten records made on our minds and hearts by the man himself.

Strong, magnetic, attractive, and, above all, self-sacrificing as a worker for the profession he loved so dearly, and for this association, which was his pride. One of the very first in conceiving the idea of its organization, and one of the foremost in working for its early life, he spared no pains and hesitated at no self-sacrifice which would forward the interests of the Southern Dental Association. His time was its time, his money its money, his thought and care were the thought and care of the organization which, frowned at by some and sneered at by others, kept alive because a few choice spirits, of whom he was one, resolved that it should not die. He was with it when in its early days it met in the Middle West, so feeble that the dentists of the city in which it assembled had to elect some of the local practitioners members before a quorum could be had. And he was with it when it stood strong without help from other societies.

Would that he were here to see the full fruition of his hopes and labors in the nearly 350 assembled here to-day.

But he is gone! We still hear, in imagination, his voice in debate and counsel; we see the kindly glance of the eye, and hear the cheery tone of the voice of the always good fellow. No differences of sentiment made him less a friend; no clashing of interests made him less enthusiastic for his calling. So strong and manly, yet so genial and kindly, that even those he fought loved him.

It is a trite saying that " He who makes two blades of grass grow where but one grew before, is a benefactor of his race," but we hold that he who tries to make one more grow, even if he fails in his endeavor, is no less a benefactor in motive and in heart. And so, if it were that no association lived to " rise up and call him blessed" as one of its fathers; had this Southern perished by frost or blight, yet could we well applaud Dr. J. R. Walker and say, " He hath done what he could."

The motive makes the man, his impulses, his character, his heart; and we reverence that, though no fruition come of all its hopes and aspirations. The " Well done, good and faithful servant" was spoken of him who was faithful over a few things.

Our friend had his convictions, and the courage to sustain them. We might not always agree with him, but they worked his individuality, and made for him a character. Enemies may snarl, but the man is there. The lesson of his life, to us, is worth learning.

He had a purpose, and tried to make circumstances aid his purpose rather than allow circumstances to mould his purpose.

We must ponder this lesson. Purpose! Circumstance! these two. Shall we drift with the tide, or take advantage of it? Circumstance without purpose is a tide to float on to destruction. Purpose, with circumstance, takes withersoever we will.

*Resolved*, That this association express its deep regret at the death of its late fellow-worker, Dr. J. R. Walker; its profound sympathy for his widow and children, and a determination that the fruits of the seed he planted with us shall have a rich harvest in the prosperity of this association.

*Resolved*, That a page of the minute-book be inscribed to his memory, and that a copy of these Resolutions be sent to his widow and children.

The resolutions were unanimously adopted.

Dr. Jas. Leslie was introduced by the President as the inventor of cohesive gold foil.

Dr. Leslie gave the history of the accidental discovery of the merits of "sticky gold" and its utilization in the first "contour" fillings, made in Louisville, Ky., in 1839, in the mouth of Dr. N. Clute.

The discussion of Dental Hygiene was resumed, and "Mrs. M. W. J.," the author of "Letters to Mothers, on the Care of Children's Teeth," was called upon. She stated briefly the results of systematic cleanliness and diet in overcoming both heredity and environment, as observed in a number of families, the results being very gratifying in the hardening of soft teeth, the arrest of decay, with fine regular dentures where, except for this special care, there was every reason to expect poor teeth.

Dr. Dunlap (Selma, Ala.) read a communication from the Alabama State Dental Society stating the action taken by that body relative to the subject of instructing the people in Dental Hygiene by means of Lectures in the Schools, Y. M. C. A.'s, etc., and asking the endorsement and co-operation of the Association.

On motion the paper was laid over for action till next year.

Dr. W. H. Richards (Knoxville, Tenn.) called the attention of the Association to the matter of the appointment of Dentists to the Army and Navy, the necessity for this having been evidenced by the condition of the teeth of the soldiers seen in the Clinics of the day previous.

Dr. W. C. Wardlaw (Agusta, Ga.) read a paper entitled

NEURALGIA: ITS ASSOCIATION WITH DENTAL LESIONS.

He spoke of the indiscriminate manner in which this term has been used, "only neuralgia" being the vague explanation of many aches and pains of which the real cause is unknown. Pain is the most distinguishing characteristic of neuralgia, the result of certain morbid conditions of the nerves, without any actual lesion, mainly from reflex irritation.

Sometimes there are complications as congestion of blood vessels, or inflammation and ulceration.

Facial neuralgia manifests itself in all the parts to which the fifth pair of nerves is distributed, fully three-fourths of the cases being, in the opinion of the writer, due to dental irritation, often from lesions so obscure in locality and character as to require most scrutinizing search to uncover them.

After considering the anatomical structure and physiological relations of the parts, the ganglionic connections, and distribution of the branches of the Fifth Pair, the writer enumerates a long list of reflex impressions, as neuralgia of the ear from an exposed pulp, several teeth aching from a single exposure, an inflamed eye from a dead pulp, etc. For earache he looks for the trouble in the lower third molar; the superior molars induce headache; lesion of the bicuspids produces pain in the cheek: he has even known neuralgia of the posterior scalp to proceed from a dead lateral.

Dr. J. J. R. Patrick (Bellville, Ill.) read a paper on the

CORRECTION OF IRREGULARITIES, illustrated on a large model and a series of charts showing the various phases of irregularities from mere "variation" from the normal type to "monstrosities." Perfect regularity is the result of the growth of all the bones with equal pace. The forty-six bones of the face all commence as jelly and finish as solid bone. If they grow with equal pace until they meet we have uniformity. If some grow too fast and others too slow, we have the different phases of irregularity. Where there are so many different parts to keep up this equal pace the wonder is that there is not a greater lack of uniformity.

The typical results of the arrest of development, or excess of development of the different separate bones, or the maxillaries, the vomer, the intermaxillary bone, the palatal processes, palatal walls, sphenoid, were all illustrated in the charts and defined in the lecture, as seen in hair lip, cleft palate, prognathism, V-shaped arch. Dr. Patrick gave special emphasis to the fact that each tooth, both deciduous and permanent, brings up its own process, and that there is no affinity as to position or place between the two sets of teeth, the idea that the deciduous teeth serve as guides to the permanent teeth being but a beautiful myth, having no foundation in fact, and also denies the existence of anything like an absorbent organ and defines the process by which the deciduous teeth are disposed of as one of *exuviation*.

In the brief discussion which followed, Dr. W. H. Atkinson took strong exception to the use of the term exuviation to designate what is really absorbtion, there being a return to embryonic conditions and the debris taken up at the point where molecular metamophosis occurs. He thought the speaker indulged too much in fanciful generalizations. He defined exuviation as the throwing off of a corneous layer of epithelium, while the lime-salts are melted and carried away by the lymphatics.

The election of officers being the next order of business, Dr. J. H. Prewitt, Madisonville, Ky., and Dr. B. H. Catching, Atlanta, Ga., were nominated candidates for the office of President.

Dr. B. H. Catching receiving a majority of all the votes cast, his election was, on motion of Dr. Prewitt, declared unanimous.

Dr. J. H. Prewitt was elected First Vice-President;

Dr. W. N. Morrison, St. Louis, Mo., Second Vice-President;

Dr. J. Hall Moore, Richmond, Va., Third Vice-President;

Dr. J. N. Crawford, Nashville, Tenn., Cor. Secretary;

Dr. L. P. Dotterer, Charleston, S. C., Rec. Secretary;

Dr. H. A. Lowrance, Athens, Ga., Treasurer.

Drs. Edwards and Doyle, Louisville, Ky., and Dr. Wm. Dancy, Jacksonville, Fla., were elected Executive Committee.

The Association then adjourned till 8 P. M. when the subject of Pathology and Therapeutics was continued.

Dr. J. McKellops, chairman of the committee on Voluntary Essays, announced a paper prepared for this meeting by the late Dr. J. R. Walker, New Orleans, La., during his last illness.

On motion, the paper was read by title:

THE FUTURE OF DENTISTRY: A PROPHECY, and ordered published in the Proceedings.

Dr. W. D. Dunlap announced the promise of a paper from Dr. Reese, of Galveston (not yet received).

Dr. Wardlaw's paper being open to discussion, Dr. Geneve, Baltimore, gave the history of a case which had been treated for neuralgia without relief, until the real cause—a dental lesion—was discovered, when a cure speedily followed.

Dr. R. Finley Hunt (Washington) differed from Dr. Wardlaw in his opinion that pure neuralgia was due to dental lesions, it being an abnormal systemic condition.

Dr. L. G. Noel (Nashville, Tenn.) read a paper entitled

### Etiology of Caries of the Teeth, Viewed from the Standpoint of Physiological Chemistry.

Referring briefly to the various theories advanced, the germ theory, the fermentation theory, etc., the writer thought that the labors of Magitot, Miller, Watt, and others might all be harmonized and combined into a grand whole, and the story told in one word—that word Catalysis.

As the diastase of fruit converts the starch of the fruit into glucose—the ptyaline in saliva producing the same result—pepsin transforming albuminous matter into peptone—all catalytic processes, so, in caries of the teeth there is a growth of catalytic substance, which, while not exerting a direct action upon the tooth tissues, is the third party bringing about the mischief by the introduction of the other two parties. In all the dejective or solvent processes there are mild acid fluids, containing a ferment—the catalytic agent. When food is dissolved in the interstices between the teeth, forming similar mild acids, cavities of decay are formed, with all the attendant phenomena of fungi, etc.

This paper was passed without discussion.

Prof. J. B. Hodgkins (Baltimore Dental College) read a paper entitled

### Are We Justified in Promising Success in Replantation?

This was the history of an unsuccessful case of replantation in which the upper central incisors, which had been knocked out by a fall, were washed in tepid carbolized water and replaced in their sockets, without removing the contents of the root canals. At the end of the first month the pulps were apparently alive. Six months later one of the incisors presented with a dead pulp, which was removed and the canal filled. Two months afterwards the teeth began to elongate and project, and in a little more than a year after replanting both teeth were removed, being very loose and the roots found greatly absorbed.

A second case was that of an implanted tooth, the operation having been performed by Dr. Younger. For a while it seemed to be firm and useful, but within two months it was removed with the fingers, the root being fairly honeycombed with cavities of absorption, looking worm-eaten. In conclusion the writer asked the question, Is it probable that a tooth, extracted and dry, with a dessicated membrane on its surface, and this a membrane never exposed with impunity in health, this membrane covering a tissue very analogous to bone, with lecunæ and canaliculi, then filled with dried and possibly decomposed protoplasmic matter, is it likely that these dry bones can live?

Dr. Morrison (St. Louis) answered Prof. Hodgkin's question in the affirmative. From his many successful cases, which far outnumbered his failures, he considered replantation in every way a legitimate operation. He thought the failure in the case cited by Prof. Hodgkins due to the fact that the great mistake had been made of not removing the contents of the root canals from the start. The whole secret of properly caring for the teeth consists in rubbing or brushing the gums as thoroughly as the teeth. This will prevent red margins and absorption in case of replantation. A replanted tooth should also be held as firmly in place, by proper splints, as a broken bone.

Dr. Stubblefield (Nashville, Tenn.) read a paper on the

### Histology of Hard Structures.

A resumé and abridgment, attempting to bring within the scope of a paper, a simplification of "the somewhat obscure, lengthy productions of the best authors."

The reading of this paper occupying the time till nearly midnight, the installation of officers was postponed until the next day, Saturday, Sept. 3, on board the steamer "Jane Morely," *en route* for Washington City, a recess being taken to enable the members to participate in the proceedings of the Dental Section of the Ninth International Medical Congress, after which the Association adjourned to meet in joint session with the American Dental Association, at Lexington, Ky., in August 1888.

